# Surgical Treatment of Falcotentorial Meningioma: An Illustrative Case Report and Literature Review

**DOI:** 10.7759/cureus.55581

**Published:** 2024-03-05

**Authors:** Roy Ferrufino-Mejia, Héctor A Rodríguez-Rubio, Rodrigo López-Rodríguez, Cesar Tomas Espinoza-Montaño, Santiago Puentes-Rosero, Kevin Sanchez-Toache, Alan Ferrufino-Mejia

**Affiliations:** 1 Neurosurgery, Mexican Institute of Social Security (IMSS) XXI Century National Medical Center, Mexico City, MEX; 2 Neurosurgery, National Institute of Neurology and Neurosurgery, Manuel Velasco Suárez, Mexico City, MEX; 3 Neurosurgery, Hospital Juárez de México, Mexico City, MEX

**Keywords:** falcotentorial junction, pineal, meningioma, surgery, falcotentorial meningioma

## Abstract

Falcotentorial meningiomas are rare tumors, representing only 2-3% of all intracranial meningiomas. These tumors can grow rapidly, leading to severe neurological complications. They grow at the junction of the tentorium cerebelli and the falx cerebri, in close proximity to the great vein of Galen. The surgical approach depends on several factors, such as the tumor's size, the patency of the straight sinus, and its location, either above or below the tentorium. Complete removal of the tumor in this area is difficult due to its deep location near major neurovascular structures. Various surgical approaches can be employed to remove these tumors, and the decision on which approach to use should be based on its advantages and disadvantages.

## Introduction

Only 3-8% of tumors in the pineal region correspond to meningiomas [1]. We can separate the pineal region meningiomas into two types according to the topographic origin, falcotentorial and velum interposition meningiomas [2]. Falcotentorial meningiomas grow at the intersection of the tentorium and the falx cerebri. Falcotentorial meningiomas only account for 1-2% of all meningiomas [3,4]. Diagnosing a pineal region tumor can be difficult due to the compression caused by meningiomas of the velum interposition [5]. Surgery to remove a tumor in a deep location near critical structures like the dural sinuses and the pineal gland can be challenging. While occipital transtentorial/transfalcine and supra cerebellar infratentorial approaches are commonly used, the approach must be chosen based on the individual case [6].

## Case presentation

A 35-year-old woman with systemic arterial hypertension is referred to a tertiary care hospital with a history of a severe paroxysmal headache of three years of evolution. For the past year, she has been experiencing nausea and vomiting, which suggests an increase in intracranial pressure. The neurological examination revealed the presence of paresis in the left lower limb and blurry vision, which was established one month ago.

**Figure 1:**
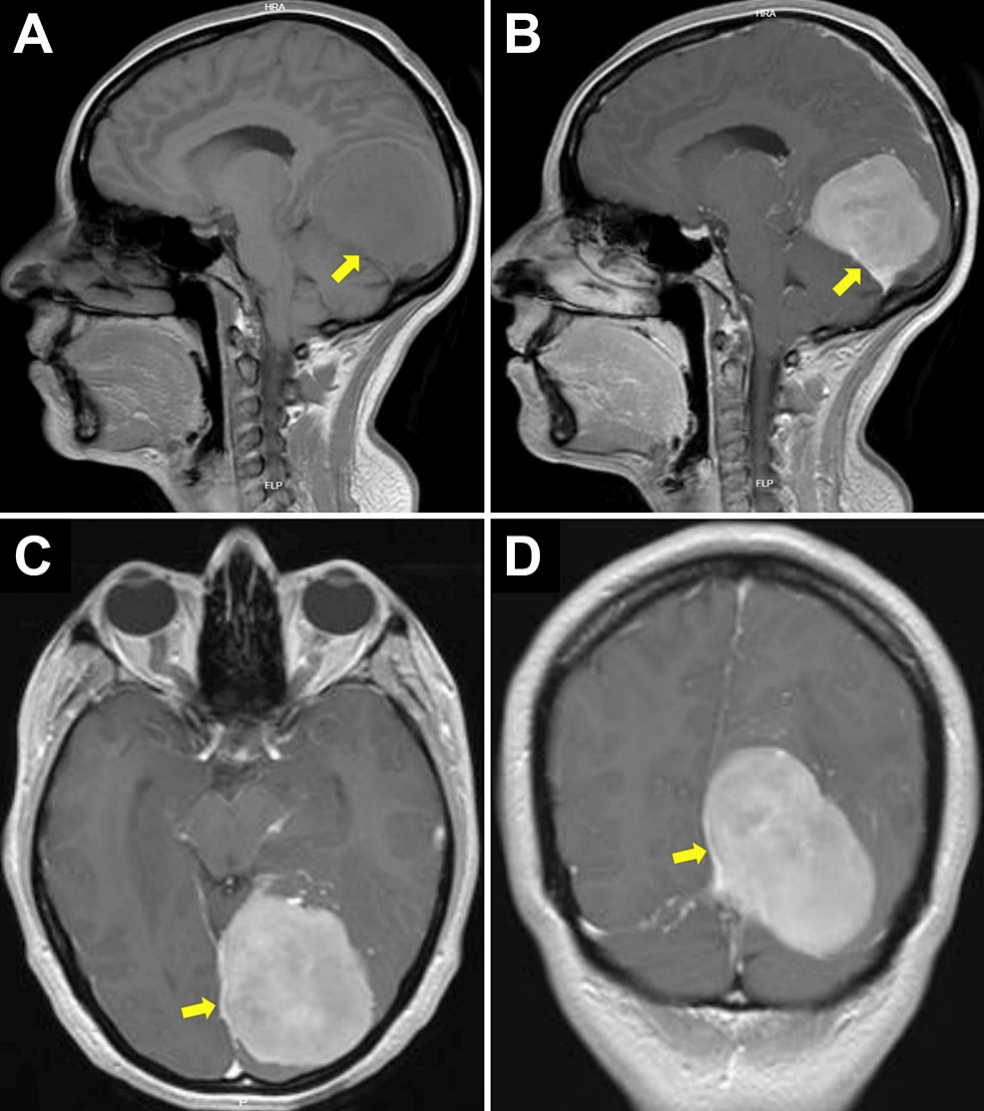
Pre-surgical MRI (Panels A, B) Sagittal MRI views. (A) Sagittal T1-weighted MRI without contrast shows an isointense lesion to gray matter. (B) Contrast-enhanced T1-weighted images show homogeneous enhancement after gadolinium administration. (Panels C, D) Axial and coronal T1-weighted MRI. They show an oval lesion with contrast enhancement of 65 mm maximum diameter, of left supratentorial location in direct relation to the medial aspect of the occipital lobe of the left cerebral hemisphere, with fixation base at the falcotentorial junction along the straight sinus (Fassiouni type IV) and with posterior extension (Bassiouni type IV) (arrows).

Preoperative MRI shows a supratentorial tumor with a maximum diameter of 65 mm, localized left parafalcine, with a base of fixation in the falcotentorial junction along the straight sinus and a posterior extension (Table 1) (Figure 1) [7].

**Table 1:** Further classification of posterior tentorial notch meningioma (TNM) in accordance with Bassiouni’s classification in 2008

Bassiouni Type	Characteristic
I	Originating between the leafs of the falx above the junction of Galen´s V with the straight sinus. Displacing the Galenic venous system inferiorly.
II	Originating from underneath the tentorium near the junction of the Galen´s V with the straight sinus. Displacing the Galenic venous system superiorly.
III	Originating from the paramedian tentorial notch. The Galen’s V lies medial to the tumor.
IV	Originating from the falcotentorial junction along the straight sinus. Displacing the Galenic venous system contralaterally.

There was no evidence of hydrocephalus. It was decided to perform a surgical procedure. The patient was positioned in the left Park Bench position; a craniotomy was performed centered on the lesion, a durotomy was carried out, and using a transtentorial/transfalcine occipital approach, the lesion, of soft consistency and rubbery appearance, was debulked with the use of an ultrasonic aspirator and the site of implantation is confirmed depending on the falcotentorial junction with posterior extension.

**Figure 2:**
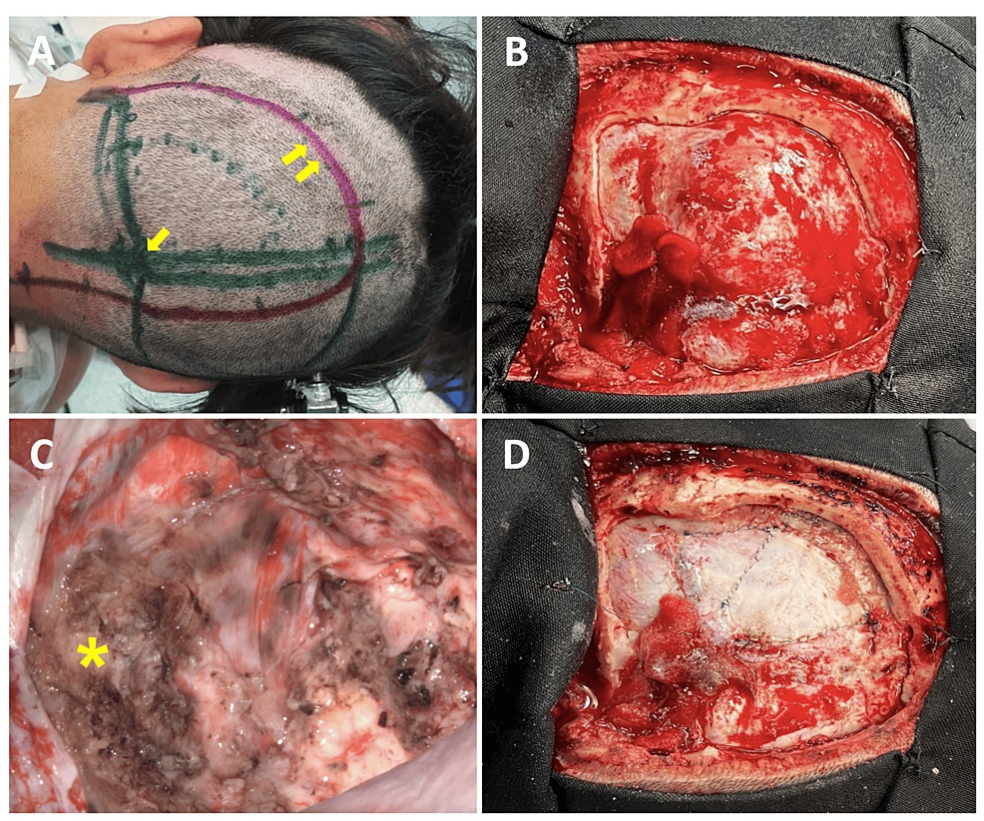
Surgical aspects (A) After positioning the patient on the Park Bench, surgical planning and demarcation of the incision by surface anatomy was performed. Green lines represent the course of the dural venous sinuses, while the yellow arrow indicates the point where the sinuses converge (arrow). Purple lines indicate the lateral course of the superior longitudinal sinus (double arrow). (B) The craniotomy performed was centered on the lesion. (C) After resection, free edges were obtained except on the medial edge, where a residual slab remains (*) due to its relationship and involvement with the torcula. (D) It was finished with the dural closure in two sheets without complications.

Once the tumor capsule was identified, we performed a Simpson III tumor excision, leaving free edges except in its medial border, where a residual slab is left due to its relation and compromise with the torcula. Hemostasis of the surgical site was continued with Gelfoam, Tissieel, and Surgicel, and the procedure was completed with anatomical plane closure (Figure 2).

**Figure 3:**
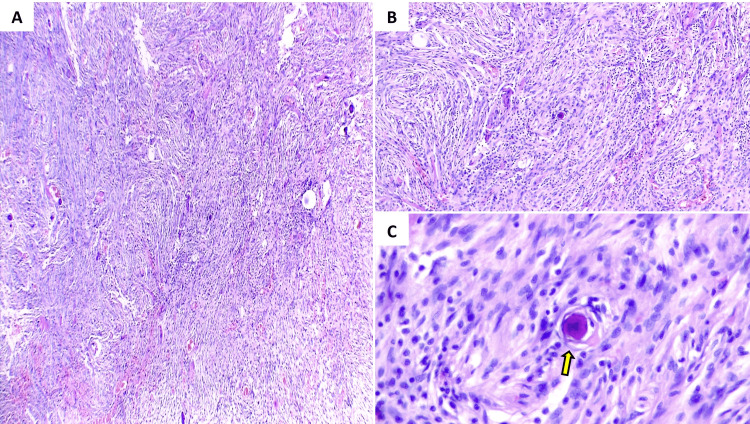
Histological sections Histologic examination stained slice showing low-grade meningothelial neoplastic lesion composed of tapered cells with elongated nuclei of compact chromatin without atypia, a psammoma body is identified between them (arrow). (A) 4x, (B) 10x, (C) 40x.

There were no complications during the surgery. According to the WHO classification, the postoperative histopathologic report revealed a grade I meningothelial hemorrhagic meningioma. The tumor had elongated cells with compact chromatin without atypia, and a psammoma body was present (Figure 3).

**Figure 4:**
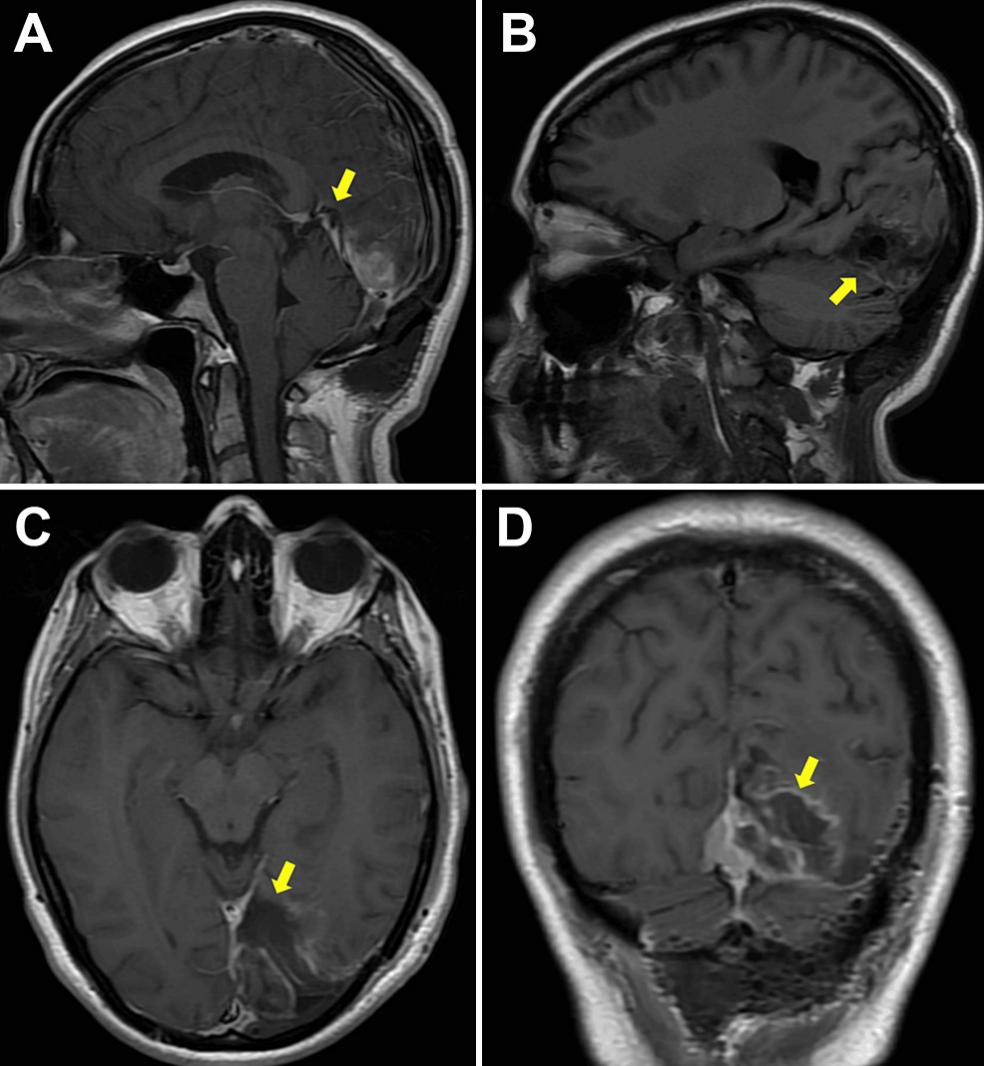
Post-surgical MRI (Panels A, B) Sagittal MRI views. (C) Axial and (D) coronal MRI views. Resection of the lesion can be observed; a residual slab remains in the medial region due to its relationship and involvement with the torcula.

After the surgery, the postoperative MRI showed that a portion of the lesion was still present in the medial region due to its relationship with the torcula. Based on the histopathologic report of grade I according to the WHO classification, expectant management and imaging control were decided to be performed in this case (Figure 4).

## Discussion

Falcotentorial meningiomas refer to a specific type of brain tumor that emerges at the convergence of the tentorium and falx cerebri. The lesions can arise at any point along it, spanning from the connection between the great vein of Galen and the straight sinus to the confluence of the sinuses. They are relatively uncommon, accounting for only 2%-3% of all meningiomas found in the brain [8]. Purely falcotentorial meningiomas are even rarer, with only 15 cases reported in the most extensive series to date, which was published by Qiu et al. in 2014 [9].

Morphological analysis is a technique that helps identify various classifications and sub-classifications of tumor types based on their histology and degree of malignancy. The World Health Organization (WHO) classification divides tumors into three grades - benign or histological grade I, atypical or grade II, and malignant or grade III. It is estimated that around 80% of identified tumors are benign (grade I), 17% are atypical (grade II), and only 3% are malignant (grade III) [10]. Based on the WHO grading system, the histopathological assessment has confirmed that it is a Grade I meningothelial hemorrhagic meningioma. Falcotentorial meningiomas can lead to various symptoms in patients, affecting the central nervous system due to their location. These symptoms include sudden and severe headaches, difficulty with movement, swelling of the optic nerve, cognitive decline, neuro-ophthalmological disorders, weakness, and, in cases where the cerebellum is significantly impacted, problems with balance and coordination [11,12].

Falcotentorial meningiomas are tumors that occur in a difficult-to-reach location and require a personalized treatment plan due to their proximity to critical anatomical structures like the pineal gland, third ventricle, and mesencephalic tectum. Removal of these tumors can be tricky because they are located near veins of the galenic group, medial posterior choroidal arteries, thalamus, and corpus callosum. With proper strategic planning, tumors can be successfully treated based on their size, location, and direction of growth [13,14]. Surgery approaches for falcotentorial meningiomas include occipital interhemispheric, supra cerebellar infratentorial, anterior interhemispheric trans splenial, occipital transtentorial, or a combination of other approaches [2,15,16].

There are two primary approaches in treating pineal or posterior third ventricular lesions: the occipital transtentorial and infratentorial supracerebellar approaches [17,18]. These approaches are typically utilized for tumors located below the deep venous complex of the pineal region. However, there have been many variations reported [7,19,20]. Surgical planning considers the angle formed by the line joining the knee's lower limit and the corpus callosum's splenium with the trajectory of the origin of the straight sinus [21,22]. When dealing with pineal lesions, there are two approaches to consider based on the angle of the lesion. If the angle is greater than 60 degrees, it is best to use the occipital-transtentorial approach. However, if the angle is less than 30 degrees, the infratentorial supracerebellar approach is preferable [19,21].

Suzuki et al. [20] removed a tumor using biparietooccipital craniotomy in the "sea-lion" position. Sekhar and Goel [21] and Ziyal et al. [23] reported a combined supra/infratentorial transsinus approach. Okada et al. [24] recommended the parietooccipital interhemispheric transfalcine and transtentorial approach for meningiomas with an anterior type (Asari). Still, it is known to cause visual impairments due to the need to retract the precuneus [18]. A combined occipital transtentorial and transfalcine corridor, recommended by Nowak et al., involves minimal risk of air embolism and good visibility of the internal cerebral vein [25].

This surgical approach is effective for treating falcotentorial meningiomas situated above and below the cerebellar tentorium. No crucial structures surround this area except for the superior sagittal and transverse sinuses [7]. During the procedure, it is crucial to avoid excessive retraction of the occipital lobe to ensure complete removal of the tumor, which can be easily achieved with this approach.

## Conclusions

Falcotentorial meningiomas are brain tumors located in a sensitive area of the brain. Due to their location, they can be difficult to treat and have the potential to cause severe damage to neurological structures. The surgical approach used to treat falcotentorial meningiomas must be customized to each patient based on the size, location, and direction of growth of the tumor; it has to be personalized to minimize complications and ensure effective tumor control. For patients, as in our case, with enlarging tumors that cause progressive neurological deficits, such as fourth cranial nerve deficits, visual deficits, and/or signs and symptoms of intracranial hypertension, the transtentorial/transfalcine occipital approach is recommended. The prognosis depends on the tumor's characterization and malignancy.

## References

[1] Blasco García de Andoain G, Delgado-Fernández J, Penanes Cuesta JR, Gil-Simoes R, Frade-Porto N, Sánchez MP (2019). Meningiomas originated at the falcotentorial region: analysis of topographic and diagnostic features guiding an optimal surgical planning. World Neurosurg.

[2] Ding Y, Sun L, Hu Y (2022). Combined microscopic and endoscopic surgery for pineal region meningiomas using the occipital-parietal transtentorial approach. Front Oncol.

[3] Dalle Ore CL, Magill ST, McDermott MW (2020). Falcotentorial meningiomas. Handb Clin Neurol.

[4] Hong CK, Hong JB, Park H, Moon JH, Chang JH, Lee KS, Park SW (2016). Surgical treatment for falcotentorial meningiomas. Yonsei Med J.

[5] Zhao X, Belykh E, Przybylowski CJ (2019). Surgical treatment of falcotentorial meningiomas: a retrospective review of a single-institution experience. J Neurosurg.

[6] Ibn Essayed W, Al-Mefty O (2022). Falcotentorial meningioma resection through the supracerebellar infratentorial approach: 2-dimensional operative video. Oper Neurosurg (Hagerstown).

[7] Bassiouni H, Asgari S, König HJ, Stolke D (2008). Meningiomas of the falcotentorial junction: selection of the surgical approach according to the tumor type. Surg Neurol.

[8] Ajler P, Beltrame S, Massa D, Tramontano J, Baccanelli M, Yampolsky C (2017). Relevance of Simpson's grades in the resection of grade I meningiomas (Article in Spanish). Surg Neurol Int.

[9] Qiu B, Wang Y, Ou S, Guo Z, Wang Y (2014). The unilateral occipital transtentorial approach for pineal region meningiomas: a report of 15 cases. Int J Neurosci.

[10] Louis DN, Perry A, Reifenberger G (2016). The 2016 World Health Organization Classification of Tumors of the Central Nervous System: a summary. Acta Neuropathol.

[11] Zingesser LH, Schechter MM (1964). The radiology of masses lying within and adjacent to the tentorial hiatus. Br J Radiol.

[12] Simon E, Afif A, M'Baye M, Mertens P (2015). Anatomy of the pineal region applied to its surgical approach. Neurochirurgie.

[13] Couldwell WT (2017). Left occipital craniotomy for resection of falcotentorial meningioma. Neurosurg Focus.

[14] Quiñones-Hinojosa A, Chang EF, Chaichana KL, McDermott MW (2009). Surgical considerations in the management of falcotentorial meningiomas: advantages of the bilateral occipital transtentorial/transfalcine craniotomy for large tumors. Neurosurgery.

[15] Talacchi A, Biroli A, Hasanbelliu A, Locatelli F (2018). Surgical management of medial tentorial meningioma: falcotentorial and torcular. World Neurosurg.

[16] Asari S, Maeshiro T, Tomita S, Kawauchi M, Yabuno N, Kinugasa K, Ohmoto T (1995). Meningiomas arising from the falcotentorial junction. Clinical features, neuroimaging studies, and surgical treatment. J Neurosurg.

[17] Konovalov AN, Spallone A, Pitzkhelauri DI (1996). Meningioma of the pineal region: a surgical series of 10 cases. J Neurosurg.

[18] Nishiura I, Handa H, Yamashita J, Suwa H (1981). Successful removal of a huge falcotentorial meningioma by use of the laser. Surg Neurol.

[19] Okami N, Kawamata T, Hori T, Takakura K (2001). Surgical treatment of falcotentorial meningioma. J Clin Neurosci.

[20] Suzuki M, Sobata E, Hatanaka M, Suzuki S, Iwabuchi T, Makiguchi K (1984). Total removal of a falcotentorial junction meningioma by biparietooccipital craniotomy in the sea lion position: a case report. Neurosurgery.

[21] Sekhar LN, Goel A (1992). Combined supratentorial and infratentorial approach to large pineal-region meningioma. Surg Neurol.

[22] Papadimitriou K, Cossu G, Rocca A, Daniel RT (2022). Occipito-transtentorial approach for falcotentorial meningiomas: how I do it. Acta Neurochir (Wien).

[23] Ziyal IM, Sekhar LN, Salas E, Olan WJ (1998). Combined supra/infratentorial-transsinus approach to large pineal region tumors. J Neurosurg.

[24] Okada T, Miyahara K, Tanino S (2020). Parieto-occipital interhemispheric transfalcine, trans-bitentorial approach for radical resection of falcotentorial meningiomas. J Neurol Surg A Cent Eur Neurosurg.

[25] Nowak A, Dziedzic T, Czernicki T, Kunert P, Marchel A (2014). Falcotentorial and velum interpositum meningiomas: two distinct entities of the pineal region. Neurol Neurochir Pol.

